# Efficacy of flossing and mouth rinsing regimens on plaque and gingivitis: a randomized clinical trial

**DOI:** 10.1186/s12903-024-03924-4

**Published:** 2024-02-03

**Authors:** Mary Lynn Bosma, James A. McGuire, Alicia DelSasso, Jeffery Milleman, Kimberly Milleman

**Affiliations:** 1 Johnson & Johnson Consumer, 199 Grandview Road, Skillman, NJ 08558 USA; 2 Salus Research, 1220 Medical Park Drive, Building 4, IN Fort Wayne, 46825 USA

**Keywords:** Mouthrinse, Alcohol-free, Plaque, Gingivitis, Essential oils, Flossing, Gingival bleeding, Dental biofilm, Oral hygiene, Brushing

## Abstract

**Background:**

To investigate the effects of combinations of mechanical (brushing and flossing) and chemotherapeutic regimens which included essential oils (EO) non-alcohol and alcohol-containing mouthrinses compared to brushing only in the prevention and reduction of plaque, gingivitis, and gingival bleeding.

**Methods:**

This was a randomized, virtually supervised, examiner blind, controlled clinical trial. Following informed consent and screening, subjects (*N* = 270) with gingivitis were randomly assigned to one of the following regimens: (1) Brush Only (B, *n* = 54); (2) Brush/Rinse (EO alcohol-containing mouthrinse) (BA, *n* = 54); (3) Brush/Rinse (EO non-alcohol containing mouthrinse) (BZ, *n* = 54); (4) Brush/Floss (BF, *n* = 54); (5) Brush/Floss/Rinse (EO non-alcohol containing mouthrinse) (BFZ, *n* = 54). Unflavored waxed dental floss (REACH unflavored waxed dental floss), and fluoridated toothpaste (Colgate Cavity Protection) were used. Examinations included oral hard and soft tissue, plaque, gingivitis, gingival bleeding, probing depth and bleeding on probing.

**Results:**

After 12 weeks, both BA and BZ and the BFZ group were superior in reducing interproximal plaque (30.8%, 18.2%, 16.0%, respectively), gingivitis (39.0%, 36.9%, 36.1%, respectively), and bleeding (67.8%, 73.6%, 79.8%, respectively) compared to B. The BF group did not provide significant reductions in interproximal plaque but did reduce interproximal gingivitis (5.1%, *p* = 0.041) at Week 4 and bleeding at Weeks 4 and 12 (34.6%, 31.4%, *p* < 0.001 respectively) compared to B. The BFZ group did not significantly reduce interproximal plaque, gingivitis or bleeding compared to BZ.

**Conclusions:**

This study demonstrated that the addition of EO non-alcohol containing mouthrinse to the manual toothbrushing and flossing regimen further reduces plaque, gingivitis and bleeding showing that addition of EO mouthrinses (alcohol or non-alcohol containing) to the oral hygiene regimen provides sustained reductions in plaque to help maintain gingival health after a dental prophylaxis. Dental professional recommendation of the addition of an EO non-alcohol containing mouthrinse to daily oral hygiene routines of brushing or brushing and flossing should be considered to aid supragingival plaque control and improve gingivitis prevention.

**Study registry number:**

NCT05600231.

## Background

Interdental cleaners, such as floss, are considered an essential part of daily oral care. The use of silk thread for interdental cleaning was first documented by a dental surgeon in the early 1800’s to be used in addition to brushing to prevent dental disease [1] and in 1898 Johnson & Johnson made silk floss widely available as a by-product of sterile silk leftover from the manufacture of sterile sutures [2]. The U.S. Food and Drug Administration specifies the characteristics and use of floss as “a string-like device made of cotton or other fibers intended to remove plaque and food particles from between the teeth to reduce tooth decay” [3].

Multiple studies have been conducted documenting the effectiveness of interproximal cleaning to prevent and control dental diseases and a 2019 Cochrane Database Systematic review concluded that “using floss or interdental brushes in addition to toothbrushing may reduce gingivitis or plaque, or both, more than toothbrushing alone” [4]. The American Dental Association (ADA) recommends that toothbrushing be performed twice daily and cleaning between teeth with floss (or another interdental cleaner) daily [5]. Other organizations, such as the National Health Service (NHS) in UK recommend the use of interdental brushes or floss as an alternative for those people with smaller spaces in between their teeth [6].

A recently published study by Milleman et al. has demonstrated that under virtual supervision, oral care regimens that included four essential oils (EOs) alcohol-containing mouthrinse (in combination with brushing or with brushing and flossing) significantly reduced supragingival plaque, gingivitis and gingival bleeding as compared to toothbrushing only or brushing and flossing after 12 weeks [7]. In that study, a virtually supervised brushing and flossing regimen was not significantly different from brushing only after 12 weeks in the reduction of supragingival plaque. The authors concluded that the results of the study provided evidence that a three-part oral hygiene regimen of brushing, flossing and rinsing with an EO alcohol-containing mouthrinse should be recommended by professionals to further assist patients in the control of plaque and gingivitis.

As the Milleman et al. study [7] tested only an EO alcohol-containing mouthrinse, further research was warranted on EO non-alcohol containing mouthrinses for those patient populations unable to use alcohol-containing mouthrinses for various reasons such as children under 12, individuals experiencing alcohol dependence, people with strong taste preferences and those holding certain religious beliefs [8]. An EO non-alcohol containing mouthrinse is manufactured and marketed by Johnson & Johnson Consumer (JJC) since 2011 to provide an alternative to consumers. A randomized controlled clinical study tested the efficacy of an EO non-alcohol containing mouthrinse in reducing plaque and gingivitis in comparison to the alcohol-containing variant [9]. Post-hoc analysis of these results indicated that the non-alcohol containing mouthrinse was as effective as the alcohol-containing mouthrinse for the control of plaque, gingivitis and gingival bleeding.

This 12-week clinical trial aimed to investigate the effects of various combinations of mechanical and chemotherapeutic regimens, encompassing both EO non-alcohol containing and EO alcohol-containing mouthrinses, in comparison to brushing only or brushing and flossing in the prevention and reduction of plaque, gingivitis and gingival bleeding.

## Methods

### Study design

This randomized, single-center, examiner-blind, parallel-group controlled clinical trial was conducted from 18 April 2022 to 25 July 2022 at Salus Research (Fort Wayne, Indiana, USA), an ADA qualified site. The study was conducted in accordance with the International Council on Harmonisation (ICH) Harmonised Tripartite Guidance for Good Clinical Practice, in agreement with the Declaration of Helsinki (2000), applicable local regulations and the ADA Seal of Acceptance Program Guidelines for Chemotherapeutic Products for Control of Gingivitis [10]. The study protocol was approved by the Institutional Ethics Committee on research involving humans (IntegReview Institutional Review Board, Austin, Texas, USA) and was retrospectively registered on clinicaltrials.gov (NCT05600231) on 31 Oct 2022 and updated per requirements in 2023. Written informed consent was obtained from all subjects.

### Subjects

Subject selection was as described in Milleman et al. [7] with the following exceptions: due to a microbiome component of the current study, subjects were required to refrain from use of probiotic drinks/supplements for one week prior to and throughout the study; and subjects were to have abstained from chemotherapeutic anti-plaque/anti-gingivitis products for four weeks prior to the start of the current study rather than two weeks as in Milleman, et al. Additionally, the age requirement was 18 years and above for this study, as compared with 18–60 years in Milleman, et al., due to COVID-19 risk factors at the time of that study. Non-emergency dental procedures during the study period were not permitted. Subjects with a history of significant adverse effects, including sensitivities or suspected allergies, following use of oral hygiene products such as toothpastes, mouth rinses and red food dye, known allergy or sensitivity or history of significant adverse effects to any of the investigational products and/or product ingredients (or other ingredients in the products) were excluded from the study. All randomized subjects (*N* = 270) provided written informed consent on a form compliant with the requirements of the Health Insurance Portability and Accountability Act. A healthy reference group (*n* = 30) was enrolled; they had only one study visit and did not receive any interventions. The healthy reference group was used as a comparator group for the microbiome component of this study (Min el al., under review).

### Interventions

At Baseline, after abstaining from oral hygiene for at least eight hours but not more than 18 h, subjects underwent an oral soft and hard tissue examination, gingivitis assessment, including marginal bleeding, pocket depth check, and bleeding on probing (BOP) measurement followed by a plaque assessment. A disclosing dye (D&C Red #28 solution [Reveal Disclosing Solution, Henry Schein Dental, Melville, NY, USA]) was used to aid in the assessment of plaque followed by rubber cap polishing with prophylaxis paste (NUPRO Prophy Past Medium Mint Without Fluoride 200/Bx, Henry Schein Dental). All eligible subjects received a complete dental prophylaxis which involved supra and subgingival scaling by hand and ultrasonic instrumentation to remove calculus followed by plaque and stain removel by rubber cap polishing with prophylaxis paste (NUPRO Prophy Paste Medium Mint Without Fluoride). To assess completeness of calculus and plaque removal, a second dental hygienist examined the subject’s teeth. An examination was performed to identify any missed calculus which was removed by scaling. Disclosing dye was used to identify remaining visual plaque which was then removed by additional polishing. Eligible subjects received a fluoridated toothpaste (Colgate Cavity Protection; Colgate-Palmolive Company, New York, USA) and a soft-bristled ADA Accepted toothbrush (Colgate classic; Colgate-Palmolive Company). The enrolled subjects (*N* = 270) were randomly assigned to one of five treatment groups:

1. a control “brush only” (B, *n* = 54) group;
2. first test group (BA, *n* = 54) received an EO alcohol-containing mouthrinse (LISTERINE COOL MINT Antiseptic EO alcohol-containing mouthrinse (ACM); JJC, Skillman, NJ, USA);
3. a second test group (BZ, *n* = 54) received an EO non-alcohol containing mouthrinse (LISTERINE COOL MINT ZERO ALCOHOL EO non-alcohol containing mouthrinse (AFM); JJC, Skillman, NJ, USA);
4. a third test group (BF, *n* = 54) received unflavored waxed dental floss (REACH unflavored waxed dental floss; JJC, Skillman, NJ, USA);
5. a fourth test group (BFZ, *n* = 54) received unflavored waxed dental floss (REACH unflavored waxed dental floss; JJC, Skillman, NJ, USA) plus an EO non-alcohol containing mouthrinse (LISTERINE ZERO EO non-alcohol containing mouthrinse (AFM);JJC, Skillman, NJ, USA).

In spite of what is recommended by various professional bodies, a review of the literature supports that adults brush on the average from approximately slightly over 30 s to slightly over 60 s [11]. Based on this review, a 60 s duration was chosen as a conservative estimate for the average adult brushing time with a manual brush. All trial products and materials were provided by the trial sponsor (JJC, Skillman, NJ, USA).

**Fig. 1:**
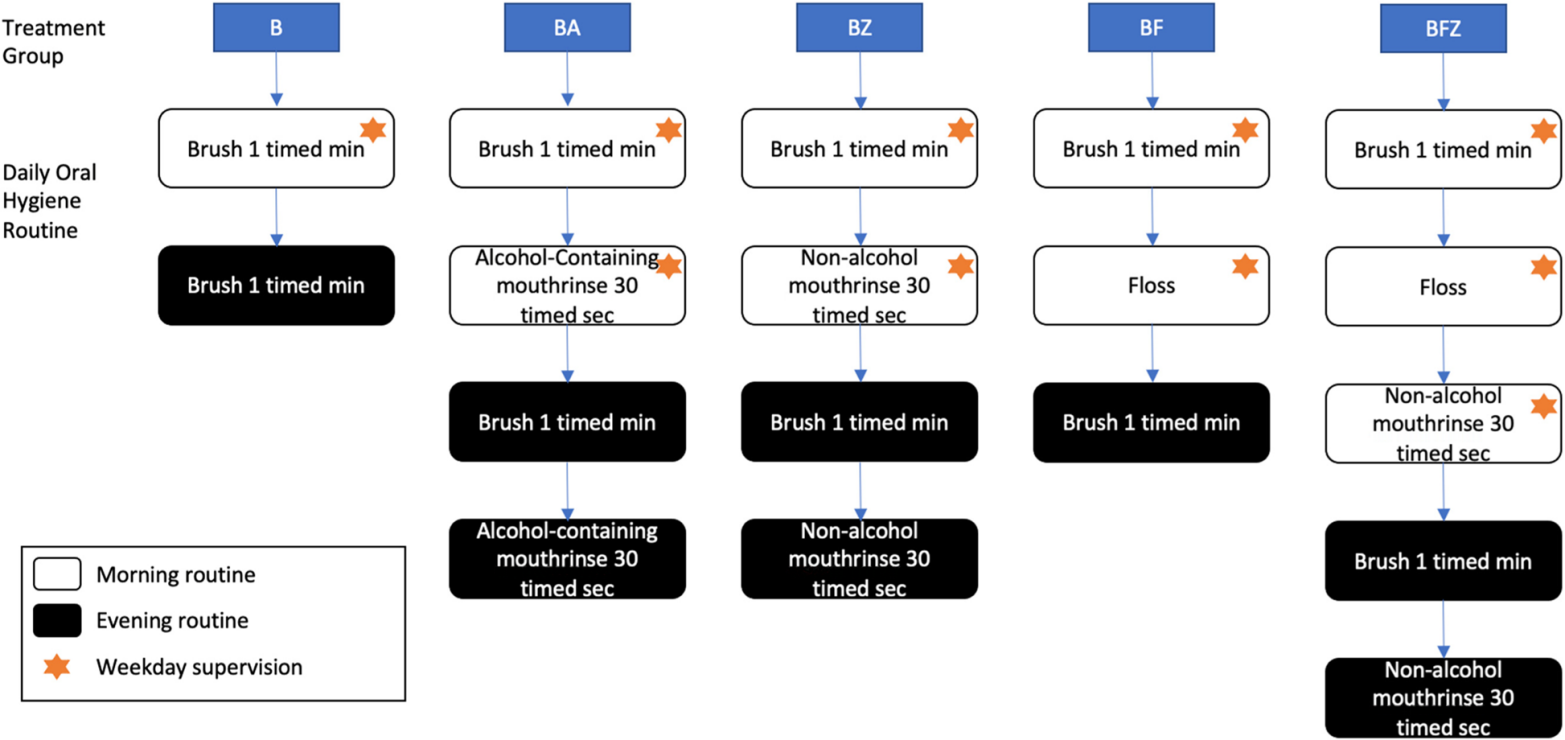
Individual treatment group daily oral hygiene routine. Subjects were supervised once in the morning on weekdays and unsupervised in the evenings or on weekends. B = Brush Only, BA = Brush/Rinse (EO alcohol-containing mouthrinse), BF = Brush/Floss, BFZ = Brush/Floss/Rinse (EO non-alcohol containing mouthrinse), BZ = Brush/Rinse (EO non-alcohol containing mouthrinse)

Both AFM and ACM contain a fixed combination of four EOs [eucalyptol (0.092%), menthol (0.042%), methyl salicylate (0.060%), and thymol (0.064%)]. At the start of the study, all subjects received a toothbrush and toothpaste and were instructed to brush with one ribbon of toothpaste in their usual manner for one timed minute twice daily. Subjects in the BF and BFZ groups received floss and were instructed in a flossing method based on the ADA-recommended technique [12] and were required to demonstrate competency. These subjects flossed following brushing during the first instance of oral hygiene daily. Subjects in the BA, BZ and BFZ groups also received the assigned mouthrinse and plastic dosage cups marked at 20 mL level and were instructed to rinse with twice daily 20 mL of full strength mouthrinse for 30 timed seconds after brushing, or brushing and flossing. All subjects received diaries to document compliance with the homecare regimen. Diaries were checked and bottles were weighed at each visit to match the number of rinses reported in the diaries to the actual used volume. Subjects performed their oral care regimen following the label instructions under supervision of study personnel for the first use in the clinic. For weekday mornings, subjects performed their oral care regimen as directed under virtual supervision via video call. They performed their oral care regimen a second time unsupervised daily in the evening and twice daily over the weekend/holidays. Consistent with label instructions on the test products, all subjects brushed twice daily. All subjects assigned to mouthrinse groups rinsed twice daily. For the two groups using dental floss, flossing was done once daily. Individual group’s daily routine is illustrated in Fig. 1.

### Randomization and blinding

Product was labeled by the clinical supplies department at the trial Sponsor with randomization numbers according to a randomization schedule generated by the trial Sponsor, using a validated program created by the Biostatistics Department at the trial sponsor, with equal allocation to treatment and block randomization with a block size of 10. Subjects were sequentially assigned a randomization number; once a randomization number was assigned to a subject, it could not be reassigned to another subject. The principal investigator (PI; JM) and examiners were blinded to the treatment regimens of the subject groups. The personnel dispensing the test products or supervising their use did not participate in the examination of subjects to minimize potential bias. Other staff members, including the PI and examiners, did not have access to the area where the products were being used.

Subjects were assessed at Baseline, Week 4, and Week 12. Assessments at Weeks 4 and 12 were made after the subjects had refrained from using their assigned product for at least eight (but not more than 18) hours and had not eaten for at least four hours. All assessment visits included a review of inclusion/exclusion criteria and concomitant medications, oral examination of hard and soft tissues, and adverse event (AE) monitoring before other measurements were taken. Each clinical index was assessed by a single, experienced, blinded and calibrated examiner. The primary indices were performed by examiners with over 25 years experience (JM, KM). BOP and pocket depth examinations were formed by a single, licensed, experienced and calibrated dental hygienist (TY). Calibration exercises were performed at the beginning of each year or within three months prior to beginning the research study. All examiners had a minimum of 90% intra-examiner Pearson correlation coefficient.

### Assessments and outcomes

Efficacy assessments were conducted at Baseline, Week 4 and Week 12 for gingivitis (Modified Gingival Index, MGI), bleeding (Expanded Bleeding Index, EBI) and plaque (Turesky Plaque Index, TPI); and probing depth and BOP at baseline and Week 12 only [7]. The primary efficacy endpoints were interproximal mean MGI and interproximal mean TPI at Week 12. Secondary endpoints included: interproximal mean TPI and MGI at Week 4; whole mouth mean TPI and MGI at Weeks 4 and 12; marginal mean TPI, MGI, and EBI at Weeks 4 and 12; whole mouth and interproximal mean bleeding index at Weeks 4 and 12; whole mouth and interproximal percent bleeding sites at Weeks 4 and 12 based on the EBI.

Gingivitis was assessed using the MGI on the buccal and lingual marginal gingiva and interdental papillae of all scorable teeth as described previously: 0 – normal (absence of inflammation); 1 – mild inflammation of any portion of the gingival unit; 2 – mild inflammation of the entire gingival unit; 3 – moderate inflammation of the gingival unit; 4 – severe inflammation of the gingival unit [9]. To assess gingival bleeding (EBI), a periodontal probe (Qulix Color Coded Probe PCP11.5B single ended, Hu-Friedy, Chicago, IL, USA) was inserted into the gingival crevice, and swept from distal to mesial around each tooth at an angle of approximately 60°, while in contact with the sulcular epithelium. Six gingival areas (distobuccal, mid-buccal, mesiobuccal, distolingual, mid-lingual, and mesiolingual) around each tooth were assessed. Bleeding at each gingival unit was recorded according to the following scale: 0 – absence of bleeding after 30 s; 1 – bleeding after 30 s; 2 – immediate bleeding [9]. A periodontal probe (Michigan O probe with Williams markings, single ended, Hu-Friedy) was used to assess pocket depth.

The plaque area, assessed using TPI, was scored on six surfaces per tooth (distobuccal, midbuccal and mesiobuccal, distolingual, midlingual and mesiolingual) of all scorable teeth as described previously: 0 – no plaque; 1 – separate flecks or discontinuous band of plaque at the gingival margin; 2 – up to 1 mm continuous band of plaque at the gingival margin; 3 – band of plaque wider than 1 mm but less than 1/3 of surface; 4 – plaque covering 1/3 or more, but less than 2/3 of surface; 5 – plaque covering 2/3 or more of surface [9].

Safety assessments included oral examinations conducted at Baseline, Week 4 and Week 12 to monitor the effect of all treatment regimens on soft and hard tissues. Changes from the Baseline and previous visits were recorded at each subsequent clinic visit. Clinically significant findings were recorded as AEs and an assessment was made regarding relationship to investigational product at the discretion of a medically qualified clinical examiner. During the study, subjects were instructed to follow their usual dietary habits and normal oral care regimen, incorporating only the toothpaste, toothbrush, floss (as appropriate) and mouthrinse (as appropriate) provided to them. No other oral hygiene procedures were permitted, including teeth cleaning or dental work, except in an emergency. Safety was assessed by summarizing all treatment emergent and treatment related AEs [9].

### Statistical analyses

This study included both non-inferiority testing and superiority testing. Sample size calculations were based only on superiority tests, as demonstrating superiority implies demonstrating non-inferiority, and consequently powering for superiority is sufficient. The planned sample size of 50 completed subjects per randomized group provided 95% power to detect a difference between BA or BZ and BF means of 0.34 for interproximal mean MGI, given a standard deviation of 0.43, based on a two-sided t-test at the 2.5% significance level. This sample size also provided greater than 99% power to detect a difference between BA or BZ and BF means of 0.54 for interproximal mean TPI, given a standard deviation of 0.38. The standard deviation estimates were based on previous three-month studies using the examiners for the current study, and the differences between means are conservative estimates based on previous studies of this type [7, 13]. Sample sizes were estimated using sample size software (PASS, version 14.0.4; NCSS, LLC, Kaysville, UT, USA).

Treatments were compared using a mixed effects model for repeated measures (MMRM), considering the within-subject covariance matrix as unstructured, and with model terms for Baseline as a covariate, treatment, visit, treatment by visit interaction and Baseline by visit interaction. For 12-week interproximal mean TPI, MGI, and EBI, and comparisons of BZ versus BF and BFZ versus BF, the familywise type I error rate was strongly controlled at 5% by separately applying a fixed sequence approach. For steps within those sequences, testing was performed at the 1.25% one-sided significance level for non-inferiority and 2.5% two-sided significance level for superiority. For BZ versus BF, non-inferiority with respect to TPI and MGI was assessed first. Provided that non-inferiority was demonstrated with respect to both MGI and TPI, superiority of BZ versus BF was tested with respect to TPI and then MGI, and subsequently non-inferiority and then superiority with respect to EBI. For BFZ versus BF, superiority was similarly tested with respect to TPI, followed by MGI and then EBI. In parallel, for comparisons for 12-week interproximal mean TPI, MGI, and EPI between BA and BF, the familywise type I error rate was strongly controlled at 5% by following the same fixed sequence approach used for BZ versus BF, but with testing at the 2.5% one-sided significance level for non-inferiority and the 5% two-sided significance level for superiority.

Non-inferiority for BZ versus BF, within the fixed sequence referenced above, was assessed by testing the null hypothesis H_01_: (µ_BZ_ - µ_B_) ≥ (1/2) (µ_BF_ - µ_B_) versus the alternative (one-sided) hypothesis H_11_: (µ_BZ_ - µ_B_) < (1/2) (µ_BF_ - µ_B_), where µ_BZ_ is the population mean for BZ and µ_B_ is the population mean for B. Rejection of H_01_ in favor of H_11_ demonstrates statistically that BZ maintains a majority of the effect of BF, where the effect of BZ is µ_BZ_ - µ_B_ and the effect of BF is µ_BF_ - µ_B_. The ratio (µ_BZ_ - µ_B_)/(µ_BF_ - µ_B_) was further explored using Fieller confidence intervals if µ_BF_ - µ_B_ was significantly different from 0. Non-inferiority for BA versus BF was tested using the same approach. (Fieller intervals are not presented in this paper, as superiority testing revealed superiority for both BZ vs. BF and BA vs. BF, and therefore exploration of the ratio (µ_BZ_ - µ_B_)/(µ_BF_ - µ_B_) and (µ_BA_ - µ_B_)/(µ_BF_ - µ_B_) was not necessary).

For each superiority test comparing BZ, BF, BFZ, and BA vs. B, the null hypothesis H_02k_: µ_k_ = µ_B_ was tested versus the alternative hypothesis H_12k_: µ_k_ ≠ µ_B_, where µ_k_ is the population mean for treatment k (= BZ, BF, BFZ, or BA) and µ_B_ is the population mean for B. For each superiority test versus BF, the null hypothesis H_03k_: µ_k_ = µ_BF_ was tested versus the alternative hypothesis H_13k_: µ_k_ ≠ µ_BF,_ where µ_k_ is the population mean for treatment k (= BZ, BFZ, or BA) and µ_BF_ is the population mean for B.

Demographic and baseline characteristics were compared across investigational product groups using Analysis of Variance (ANOVA) or a Chi-Square test or Fisher’s exact test. A statistical software package (SAS Version 9.4 software; SAS institute, Cary, NC, USA) was used for statistical analyses.

## Results

**Fig. 2:**
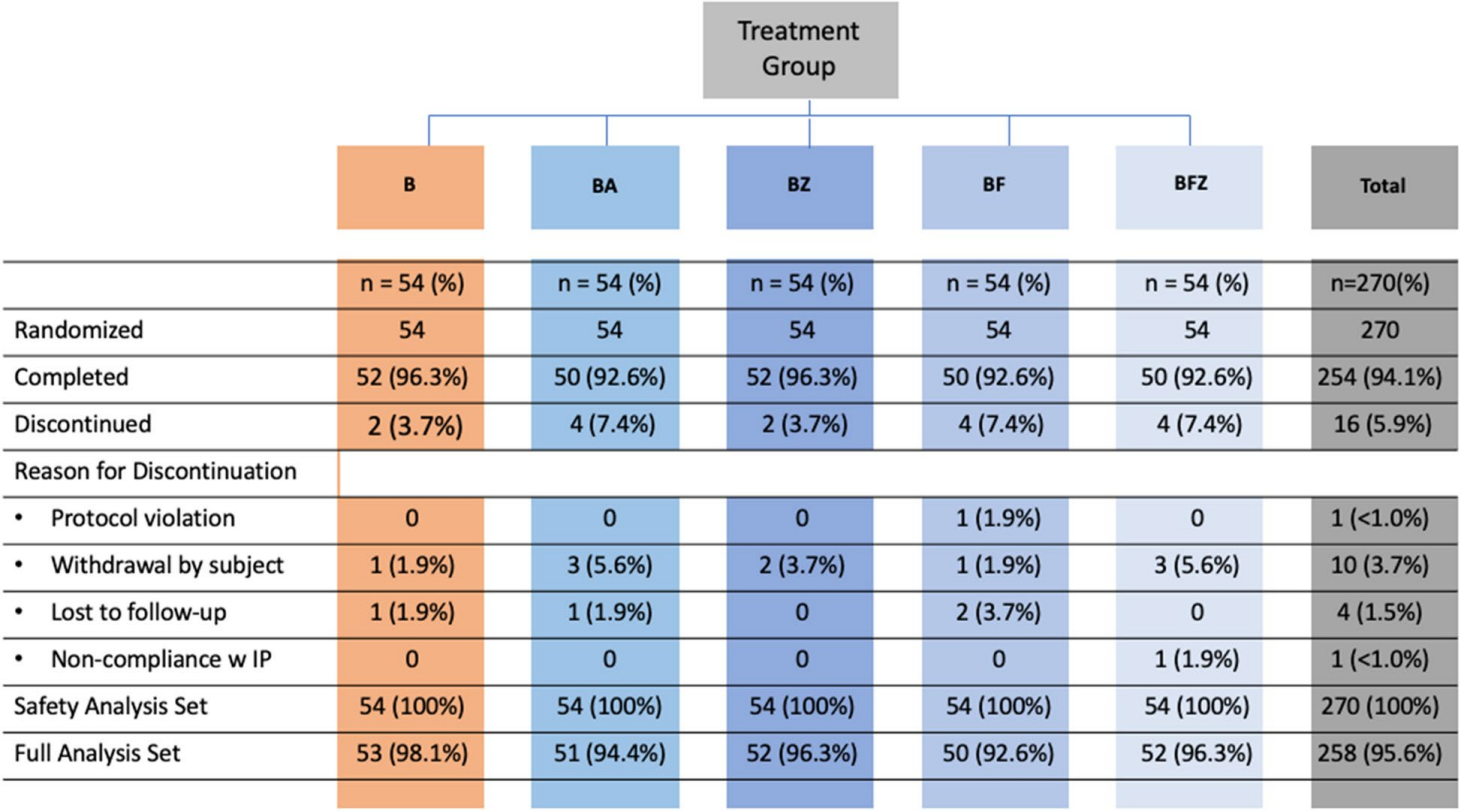
Flow chart of trial group assignments. B = Brush Only, BA = Brush/Rinse (EO alcohol-containing mouthrinse), BF = Brush/Floss, BFZ = Brush/Floss/Rinse (EO non-alcohol containing mouthrinse), BZ = Brush/Rinse (EO non-alcohol containing mouthrinse)

Of the 270 randomized subjects, 254 completed the trial. Ten subjects withdrew their consent, four were lost to follow-up, one was discontinued due to protocol violations, and one was discontinued for non-compliance. Trial group distribution is shown in Fig. 2. The sample demographics and Baseline gingival health characteristics are presented in Table 1. There were no significant differences among the groups for any demographic data or for any average baseline data for all measurements.

**Table 1:** Demographics by group assignment

Group	B	BA	BZ	BF	BFZ	Total (*N* = 270)	*p*-value
n	54	54	54	54	54	270	
Mean age (SD)	42.4 (15.85)	43.3 (15.16)	44.2 (12.30)	43.4 (13.41)	41.1 (13.76)	42.9 (14.08)	0.840^a^
Sex, n (%)							
Male	11 (20.4%)	7 (13.0%)	16 (29.6%)	13 (24.1%)	16 (29.6%)	63 (23.3%)	0.205^b^
Female	43 (79.6%)	47 (87.0%)	38 (70.4%)	41 (75.9%)	38 (70.4%)	207 (76.7%)	
Race, n (%)							
White	48 (88.9%)	47 (87.0%)	50 (92.6%)	47 (87.0%)	47 (87.0%)	239 (88.5%)	0.839^c^
Black/African American	5 (9.3%)	7 (13.0%)	4 (7.4%)	7 (13.0%)	7 (13.0%)	30 (11.1%)	
Asian	1 (1.9%)	0	0	0	0	1 (< 1.0%)	
Ethnicity, n (%)							
Hispanic/Latino	3 (5.6%)	2 (3.7%)	1 (1.9%)	1 (1.9%)	4 (7.4%)	11 (4.1%)	0.646^c^
Not Hispanic/Latino	51 (94.4%)	52 (96.3%)	53 (98.1%)	53 (98.1%)	50 (92.6%)	259 (95.9%)	
Smoker, n (%)							
No	53 (98.1%)	52(96.3%)	54 (100%)	54 (100%)	51 (94.4%)	264 (97.8%)	0.317^c^
Yes	1 (1.9%)	2 (3.7%)	0	0	3 (5.6%)	6 (2.2%)	
Baseline scores (whole mouth)							
Mean MGI (SD)	2.57 (0.310)	2.57 (0.319)	2.66 (0.287)	2.59 (0.281)	2.64 (0.263)	2.60 (0.293)	0.336^a^
Mean TPI (SD)	3.08 (0.413)	3.00 (0.463)	3.03 (0.393)	3.13 (0.441)	3.04 (0.438)	3.06 (0.430)	0.560^a^
Mean EBI (SD)	0.324 (0.2229)	0.312 (0.1761)	0.360 (0.1930)	0.346 (0.1740)	0.325 (0.1969)	0.334 (0.1927)	0.706^a^
Mean Bleeding Upon Probing Pocket Depth (SD)	0.110 (0.1535)	0.099 (0.1145)	0.138 (0.1824)	0.089 (0.1112)	0.111 (0.1298)	0.109 (0.1408)	0.444^a^
Mean Pocket Depth (SD)	2.07 (0.341)	2.00 (0.332)	2.13 (0.395)	2.06 (0.310)	2.08 (0.370)	2.07 (0.351)	0.442^a^
Baseline Scores (interproximal)							
Mean MGI (SD)	2.74 (0.269)	2.73 (0.285)	2.80 (0.224)	2.75 (0.233)	2.79 (0.207)	2.76 (0.245)	0.432^a^
Mean TPI (SD)	3.21 (0.390)	3.15 (0.409)	3.17 (0.358)	3.27 (0.400)	3.19 (0.393)	3.20 (0.390)	0.516^a^
Mean EBI (SD)	0.323 (0.2308)	0.304 (0.1846)	0.360 (0.2049)	0.344 (0.1750)	0.318 (0.1970)	0.330 (0.1989)	0.610^a^
Mean Bleeding Upon Probing Pocket Depth (SD)	0.121 (0.1745)	0.106 (0.1248)	0.151 (0.1973)	0.100 (0.1233)	0.118 (0.1408)	0.119 (0.1547)	0.490^a^
Mean Pocket Depth (SD)	2.33 (0.347)	2.24 (0.346)	2.39 (0.403)	2.30 (0.316)	2.32 (0.376)	2.32 (0.359)	0.299^a^

*B* Brush Only, *BA* Brush/Rinse (EO alcohol-containing mouthrinse), *BF* Brush/Floss, *BFZ *Brush/Floss/Rinse (EO non-alcohol containing mouthrinse), *BZ* Brush/Rinse (EO non-alcohol containing mouthrinse), *EBI* expanded bleeding index, *MGI* modified gingival index, *SD* standard deviation, *TPI* Turesky Plaque Index

^a^*p*-values are based on ANOVA model with term for treatment group

^b^*p*-values are based on Chi-Squares test

^c^Twenty% or more cells with expected cell size < 5, Chi-Square test may not be valid test. Fisher’s Exact text was used

### Primary efficacy endpoint: interproximal mean TPI and MGI at 12 weeks

Compared to the B group, the interproximal mean TPI was statistically significantly reduced at Week 12 for the three groups that included a mouthrinse (BA: 30.8%; BZ: 18.2%; BFZ: 16.0%; *p* < 0.001 for each comparison). The BF group was not statistically significantly different from B group at Week 12. BA, BZ, and BFZ groups all had statistically significant reductions in interproximal mean TPI compared to the BF group (*p* < 0.001 for each comparison), while BFZ had no statistically significant reduction compared to the BZ group (Table 2). At Week 12, the three mouthrinse groups had reduced interproximal mean MGI (BA: 39.0%; BZ: 36.9%; BFZ: 36.1%;*p* < 0.001 for each comparison) compared to B, while the BF group mean MGI reduction was not statistically significantly different compared to B group. BA, BZ, and BFZ groups all had statistically significant reductions in interproximal mean MGI versus the BF group (*p* < 0.001 for each comparison) at Week 12. BFZ did not have a statistically significant reduction in MGI at Week 12 compared to BZ group (Table 3).

**Table 2:** Interproximal mean Turesky Plaque Index (TPI) at Baseline, Weeks 4, 12

Group	B	BA	BZ	BF	BFZ
Baseline					
n	53	51	52	50	52
Mean	3.20	3.13	3.14	3.28	3.18
(SD)	0.382	0.413	0.329	0.369	0.386
Week 4					
N	53	51	52	50	52
LSmean (SE)	3.08 (0.043)	2.23 (0.044)	2.53 (0.043)	3.05 (0.044)	2.56 (0.043)
Treatment groupss versus B					
*p-*value		< 0.001	< 0.001	0.696	< 0.001
Difference (SE)		-0.85 (0.061)	-0.55 (0.061)	-0.02 (0.061)	-0.51 (0.061)
95% CI		[-0.97, -0.73]	[-0.67, -0.43]	[-0.14, 0.10]	[-0.63, -0.39]
% reduction		27.5	17.9	0.8	16.7
Brush/Rinse groups versus BF Superiority					
*p-*value Difference (SE) 95% CI % reduction		< 0.001 -0.82 (0.062) [-0.94, -0.70] 26.9	< 0.001 -0.53 (0.062) [-0.65, -0.40] 17.2		
BFZ group versus BF Superiority					
*p-*value Difference (SE) 95% CI % reduction					< 0.001 -0.49 (0.062) [-0.61, -0.37] 16.1
BFZ versus BZ					
*p-*value Difference (SE) 95% CI % reduction					0.557 0.04 (0.061) [-0.08, 0.16] -1.4
Week 12					
n	52	50	52	50	50
LSmean (SE)	2.97 (0.042)	2.05 (0.043)	2.43 (0.042)	3.05 (0.043)	2.49 (0.042)
Treatment groups versus B					
*p*-value		< 0.001	< 0.001	0.164	< 0.001
Difference (SE)		-0.91 (0.059)	-0.54 (0.059)	0.08 (0.060)	-0.48 (0.059)
95% CI		[-1.03, -0.80]	[-0.66, -0.42]	[-0.03, 0.20]	[-0.59, -0.36]
% reduction		30.8	18.2	-2.8	16.0
BZ Rinse versus BF Non-inferiority					
*p-*value			< 0.001		
BA versus BF Non-inferiority					
*p-*value		< 0.001			
BA versus BF Superiority					
*p-*value Difference (SE) 95% CI % reduction		< 0.001 -1.00 (0.061) [-1.11, -0.88] 32.6			
BZ versus BF Superiority					
*p-*value Difference (SE) 95% CI % reduction			< 0.001 -0.62 (0.060) [-0.76, -0.49] 20.4		
BFZ versus BF Superiority					
*p-*value Difference (SE) 97.5% CI % reduction					< 0.001 -0.56 (0.060) [-0.69, -0.42] 18.3
BFZ versus BZ Superiority					
*p-*value Difference (SE) 95% CI % reduction					0.278 0.065 (0.059) [-0.05, 0.18] -2.7

*B* Brush Only, *BA* Brush/Rinse (EO alcohol-containing mouthrinse), *BF* Brush/Floss, *BFZ* Brush/Floss/Rinse (EO non-alcohol containing mouthrinse), *BZ* Brush/Rinse (EO non-alcohol containing mouthrinse), *CI* confidence interval, *LSmean* least square means, *SD* standard deviation, *SE* standard error

All *p*-values, model-based estimated means (LSmeans), and standard errors were based on a mixed effects model for repeated measures analysis (MMRM), with the fixed effects including treatment, visit, and treatment by visit interaction; baseline as a covariate; and baseline by visit interaction

**Table 3:** Interproximal mean Modified Gingival Index (MGI) at Baseline, Weeks 4, 12

Group	B	BA	BZ	BF	BFZ
Baseline					
n	53	51	52	50	52
Mean	2.74	2.72	2.80	2.74	2.79
(SD)	0.270	0.286	0.226	0.225	0.208
Week 4					
N	53	51	52	50	52
LSmean (SE)	2.73 (0.047)	1.94 (0.048)	1.93 (0.048)	2.59 (0.049)	1.88 (0.048)
Treatment groups versus B					
*p-*value		< 0.001	< 0.001	0.041	< 0.001
Difference (SE)		-0.79 (0.068)	-0.80 (0.067)	-0.14 (0.068)	-0.85 (0.067)
95% CI		[-0.93, -0.66]	[-0.93, -0.67]	[-0.27, -0.01]	[-0.98, -0.72]
% reduction		29.0	29.3	5.1	31.2
Brush/Rinse groups versus BF Superiority					
*p-*value Difference (SE) 95% CI % reduction		< 0.001 -0.65 (0.069) [-0.79, -0.52] 25.2	< 0.001 -0.66 (0.068) [-0.80, -0.53] 25.5		
BFZ versus BF Superiority					
*p-*value Difference (SE) 95% CI % reduction					< 0.001 -0.71 (0.068) [-0.85, -0.58] 27.5
BFZ versus BZ					
*p-*value Difference (SE) 95% CI % reduction					0.462 -0.05 (0.068) [-0.18, 0.803] 2.6
Week 12					
n	52	50	52	50	50
LSmean (SE)	2.67 (0.052)	1.63 (0.053)	1.68 (0.053)	2.55 (0.053)	1.71 (0.053)
Treatment groups versus B					
*p*-value		< 0.001	< 0.001	0.111	< 0.001
Difference (SE)		-1.04 (0.075)	-0.99 (0.074)	-0.12 (0.074)	-0.96 (0.075)
95% CI		[-1.19, -0.89]	[-1.13, -0.89]	[-0.27, 0.03]	[-1.11, -0.82]
% reduction		39.0	36.9	4.5	36.1
BZ versus BF Non-inferiority					
*p-*value Difference			< 0.001		
BA versus BF Non-inferiority					
*p-*value		< 0.001			
BA versus BF Superiority					
*p-*value Difference (SE) 95% CI % reduction		< 0.001 -0.92 (0.075) [-1.07, -0.77] 36.2			
BZ versus BF Superiority					
*p-*value Difference (SE) 95% CI % reduction			< 0.001 -0.87 (0.075) [-1.04, -0.70] 34.0		
BFZ versus BF Superiority					
*p-*value Difference (SE) 97.5% CI % reduction					< 0.001 -0.84 (0.076) [-1.01, -0.67] 33.1
BFZ versus BZ Superiority					
*p-*value Difference (SE) 95% CI % reduction					0.770 0.02 (0.075) [-0.13, 0.17] -1.3

*B* Brush Only, *BA* Brush/Rinse (EO alcohol-containing mouthrinse), *BF* Brush/Floss, *BFZ* Brush/Floss/Rinse (EO non-alcohol containing mouthrinse), *BZ* Brush/Rinse (EO non-alcohol containing mouthrinse), *CI* confidence interval, *LSmean* least square means, *SD* standard deviation, *SE* standard error

All *p*-values, model-based estimated means (LSmeans), and standard errors were based on a mixed effects model for repeated measures analysis (MMRM), with the fixed effects including treatment, visit, and treatment by visit interaction; baseline as a covariate; and baseline by visit interaction

### Secondary efficacy endpoint: interproximal mean TPI and MGI at 4 weeks

The interproximal mean TPI was statistically significantly reduced for the three groups that included a mouthrinse at Week 4 compared to the B group (BA: 27.5%; BZ: 17.9%; BFZ: 16.7% versus the B group; *p* < 0.001 for each comparison). The BF group was not statistically significantly different from B for Week 4. Compared to the BF group, the three treatment groups using a mouthrinse had statistically significant reductions in interproximal mean TPI at Week 4 (*p* < 0.001 for each comparison) (Table 2). The interproximal mean MGI was statistically significantly reduced for all groups at Week 4 (BA: 29.0%; BZ: 29.3%; BFZ: 31.2%, *p* < 0.001 for each comparison; and BF: 5.1%, *p* = 0.041) compared to B. Compared to the BF group, the three treatment groups using a mouthrinse had statistically significant reductions in interproximal mean MGI at Week 4 (*p* < 0.001 for each comparison ) (Table 3).

### Secondary efficacy endpoint: interproximal mean EBI and percent bleeding sites at Week 4 and Week 12

Compared to the B group, interproximal mean EBI was statistically significantly reduced at Weeks 4 and 12 for all treatment groups: BA: 70.0% and 67.8%, respectively; BZ: 70.8% and 73.6%, respectively; BFZ: 77.7% and 79.8%, respectively; and BF: 34.6% and 31.4% respectively (*p* < 0.001 for each comparison). Similarly, interproximal mean EBI was statistically significantly reduced for the three treatment groups using a mouthrinse at Weeks 4 and 12 compared to BF group (Table 4). Percent bleeding sites were statistically significantly reduced for all treatment groups at Weeks 4 and 12 compared to both B and BF groups (*p* < 0.001 for each comparison). Percent reductions are shown in Table 5.

**Table 4:** Interproximal mean Expanded Bleeding Index (EBI) at Baseline, Weeks 4, 12

Group	B	BA	BZ	BF	BFZ
Baseline					
n	53	51	52	50	52
Mean	0.305	0.296	0.367	0.342	0.311
(SD)	0.1910	0.1783	0.2061	0.1756	0.1856
Week 4					
N	53	51	52	50	52
LSmean (SE)	0.213 (0.0098)	0.064 (0.0100)	0.062 (0.0100)	0.139 (0.0101)	0.047 (0.0099)
Treatment groups versus B					
*p-*value		< 0.001	< 0.001	< 0.001	< 0.001
Difference (SE)		-0.149 (0.0140)	-0.151 (0.0140)	-0.074 (0.0141)	-0.165 (0.0139)
95% CI		[-0.177, -0.122]	[-0.178, -0.123]	[-0.101, -0.046]	[-0.193, -0.138]
% reduction		70.0	70.8	34.6	77.7
Brush/Rinse groups versus BF Superiority					
*p-*value Difference (SE) 95% CI % reduction		< 0.001 -0.076 (0.0143) [-0.104, -0.047] 54.2	< 0.001 -0.077 (0.0142) [-0.105, -0.049] 55.4		
BFZ group versus BF Superiority					
*p-*value Difference (SE) 95% CI % reduction					< 0.001 -0.092 (0.0142) [-0.120, -0.064] 65.9
BFZ versus BZ					
*p-*value Difference (SE) 95% CI % reduction					0.299 -0.015 (0.0141) [-0.042, -0.013] 23.5
Week 12					
n	52	50	52	50	50
LSmean (SE)	0.241 (0.0123)	0.078 (0.0126)	0.063 (0.0124)	0.165 (0.0126)	0.049 (0.0125)
Treatment groups versus B					
*p*-value		< 0.001	< 0.001	< 0.001	< 0.001
Difference (SE)		-0.163 (0.0176)	-0.177 (0.0175)	-0.076 (0.0176)	-0.192 (0.0176)
95% CI		[-0.198, -0.129]	[-0.212, -0.143]	[-0.110, -0.041]	[-0.226, -0.157]
% reduction		67.8	73.6	31.4	79.8
BZ versus BF Non-inferiority					
*p-*value			< 0.001		
BA versus BF Non-inferiority					
*p-*value		< 0.001			
BA versus BF Superiority					
*p-*value Difference (SE) 95% CI % reduction		< 0.001 -0.087 (0.0178) [-0.122, -0.052] 53.0			
BZ versus BF Superiority					
*p-*value Difference (SE) 95% CI % reduction			< 0.001 -0.102 (0.0176) [-0.141, -0.062] 61.6		
BFZ versus BF Superiority					
*p-*value Difference (SE) 97.5% CI % reduction					< 0.001 -0.116 (0.0178) [-0.156, -0.076] 70.5
BFZ versus BZ Superiority					
*p-*value Difference (SE) 95% CI % reduction					0.406 -0.015 (0.0176) [-0.049, 0.020] 23.2

*B* Brush Only, *BA* Brush/Rinse (EO alcohol-containing mouthrinse), *BF* Brush/Floss, *BFZ* Brush/Floss/Rinse (EO non-alcohol containing mouthrinse), *BZ* Brush/Rinse (EO non-alcohol containing mouthrinse), *CI* confidence interval, *LSmean* least square means, *SD* standard deviation, *SE* standard error

All *p*-values, model-based estimated means (LSmeans), and standard errors were based on a mixed effects model for repeated measures analysis (MMRM), with the fixed effects including treatment, visit, and treatment by visit interaction; baseline as a covariate; and baseline by visit interaction

**Table 5:** Interproximal percent gingival bleeding sites at Baseline, Weeks 4 and 12

Group	B	BA	BZ	BF	BFZ
Baseline					
n	53	51	52	50	52
Mean	25.0	24.6	29.0	26.7	24.9
(SD)	12.03	12.66	13.20	10.77	11.31
Week 4					
N	53	51	52	50	52
LSmean (SE)	18.4 (0.78)	5.2 (0.80)	5.5 (0.79)	12.1 (0.80)	4.1 (0.79)
Treatment groups versus B					
*p-*value		< 0.001	< 0.001	< 0.001	< 0.001
Difference (SE)		-13.1 (1.11)	-12.8 (1.11)	-6.3 (1.12)	-14.3 (1.11)
95% CI		[-15.3, -10.9]	[-15.0, -10.6]	[-8.5, -4.1]	[-16.5, -12.1]
% reduction		71.6	69.8	34.1	77.9
Brush/Rinse groups versus BF Superiority					
*p-*value Difference (SE) 95% CI % reduction		< 0.001 -6.9 (1.13) [-9.1, -4.7] 56.8	< 0.001 -6.5 (1.12) [-8.8, -4.3] 54.1		
BFZ versus BF Superiority					
*p-*value Difference (SE) 95% CI % reduction					< 0.001 -8.0 (1.12) [-10.2, -5.8] 66.5
BFZ Rinse group versus BZ					
*p-*value Difference (SE) 95% CI % reduction					0.184 -1.5 (1.12) [-3.7, 0.7] 26.9
Week 12					
n	52	50	52	50	50
LSmean (SE)	20.2 (0.97)	6.6 (0.99)	5.7 (0.97)	15.1 (0.99)	4.4 (0.99)
Treatment groups versus B					
*p*-value^*^		< 0.001	< 0.001	< 0.001	< 0.001
Difference (SE)		-13.5 (1.38)	-14.4 (1.38)	-5.1 (1.38)	-15.8 (1.38)
95% CI		[-16.2, -10.8]	[-17.1, -11.7]	[-7.8, -2.4]	[-18.5, -13.1]
% reduction		67.0	71.5	25.2	78.4
Brush/Rinse groups versus BF Superiority					
*p-*value Difference (SE) 95% CI % reduction		< 0.001 -8.4 (1.40) [-11.2, -5.7] 55.9	< 0.001 -9.3 (1.39) [-12.1, -6.6] 61.9		
BFZ versus BF Superiority					
*p-*value Difference (SE) 97.5% CI % reduction					< 0.001 -10.7 (1.40) [-13.5, -8.0] 71.0
BFZ versus BZ Superiority					
*p-*value Difference (SE) 95% CI % reduction					0.321 -1.4 (1.39) [-4.1, 1.4] 24.1

*B* Brush Only, *BA* Brush/Rinse (EO alcohol-containing mouthrinse), *BF* Brush/Floss, *BFZ *Brush/Floss/Rinse (EO non-alcohol containing mouthrinse), *BZ* Brush/Rinse (EO non-alcohol containing mouthrinse), *CI* confidence interval, *LSmean* least square means, *SD* standard deviation, *SE* standard error

All *p*-values, model-based estimated means (LSmeans), and standard errors were based on a mixed effects model for repeated measures analysis (MMRM), with the fixed effects including treatment, visit, and treatment by visit interaction; baseline as a covariate; and baseline by visit interaction

### Secondary efficacy endpoint: interproximal mean probing depth and BOP at Week 12

All test groups showed statistically significant reductions in interproximal mean probing depth compared to the B group (*p* < 0.001 for each comparison) (Table 6). There were no statistically significant differences in interproximal mean probing depth for BF vs. BA (*p* = 0.950) or BF vs. BZ (*p* = 0.803). The BFZ group was not statistically significantly different from BF (*p* = 0.348) or BZ (*p* = 0.232) for interproximal mean probing depth at Week 12. Compared to the B group, only the BF group had a statistically significant reductions in BOP (*p* = 0.022). Interproximal mean BOP was not statistically significantly different for BF vs. BA (*p* = 0.141), however, BF provided a statistically significant reduction vs. BZ (*p* = 0.017). The BFZ group was not statistically significantly different from BF (*p* = 0.433) or BZ (*p* = 0.107) for interproximal mean BOP at Week 12 (Table 6).

**Table 6:** Whole mouth, interproximal mean probing depth and bleeding on probing (BOP), Week 12

Group	B	BA	BZ	BF	BFZ
Whole Mouth Mean Probing Depth					
Baseline					
n	53	51	52	50	52
Mean	2.05	2.00	2.12	2.05	2.07
(SD)	0.319	0.329	0.395	0.307	0.334
Week 12					
N	52	50	52	50	50
LSmean (SE)	2.02 (0.036)	1.62 (0.037)	1.64 (0.036)	1.61 (0.037)	1.57 (0.037)
Treatment groups versus B					
*p-*value		< 0.001	< 0.001	< 0.001	< 0.001
Difference (SE)		-0.40 (0.051)	-0.38 (0.051)	-0.41 (0.051)	-0.44 (0.051)
95% CI		[-0.50, -0.30]	[-0.48, -0.28]	[-0.51, -0.31]	[-0.54, -0.34]
% reduction		19.9	18.7	20.3	22.0
Brush/Rinse groups versus BF					
*p-*value Difference (SE) 95% CI % reduction		0.869 0.01 (0.052) [-0.09, 0.11] -0.5	0.541 0.03 (0.051) [-0.07, 0.13] -2.0		
BFZ versus BF					
*p-*value Difference (SE) 95% CI % reduction					0.512 -0.03 (0.052) [-0.14, 0.07] 2.1
BFZ versus BZ					
*p-*value Difference (SE) 95% CI % reduction					0.203 -0.07 (0.051) [-0.17, 0.04] 4.0
Whole Mouth Mean BOP					
Baseline					
n	53	51	52	50	52
Mean	0.099	0.099	0.144	0.087	0.106
(SD)	0.1303	0.1152	0.1840	0.1143	0.1224
Week 12					
n	52	50	52	50	50
LSmean (SE)	0.045 (0.0080)	0.034 (0.0082)	0.045 (0.0081)	0.020 (0.0082)	0.027 (0.0082)
Treatment groups versus B					
*p*-value		0.343	0.967	0.028	0.120
Difference (SE)		-0.011 (0.0114)	-0.000 (0.0114)	-0.025 (0.0115)	-0.018 (0.0114)
95% CI		[-0.033, 0.012]	[-0.023, 0.022]	[-0.048, -0.003]	[-0.040, 0.005]
% reduction		24.0	1.1	55.7	39.4
Brush/Rinse groups versus BF					
*p-*value Difference (SE) 95% CI % reduction		0.215 0.014 (0.0116) [-0.008, 0.037] -71.7	0.033 0.025 (0.0115) [0.002, 0.048] -123.5		
BFZ versus BF					
*p-*value Difference (SE) 95% CI % reduction					0.524 0.007 (0.0116) [-0.015, 0.030] -36.8
BFZ versus BZ					
*p-*value Difference (SE) 95% CI % reduction					0.132 -0.017 (0.0115) [-0.040, 0.005] 38.8
Interproximal Mean Probing Depth					
Baseline					
n	53	51	52	50	52
Mean	2.31	2.24	2.39	2.29	2.31
(SD)	0.328	0.344	0.404	0.312	0.340
Week 12					
n	52	50	52	50	50
LSmean (SE)	2.27 (0.041)	1.83 (0.042)	1.85 (0.041)	1.84 (0.042)	1.78 (0.042)
Treatment groups versus B					
*p-*value		< 0.001	< 0.001	< 0.001	< 0.001
Difference (SE)		-0.44 (0.059)	-0.42 (0.058)	-0.44 (0.059)	-0.49 (0.059)
95% CI		[-0.56, -0.33]	[-0.54, -0.31]	[-0.55, -0.32]	[-0.61, -0.38]
% reduction		19.4	18.6	19.3	21.7
Brush/Rinse groups versus BF					
*p-*value Difference (SE) 95% CI % reduction		0.950 -0.00 (0.059) [-0.12, 0.11] 0.2	0.803 0.02 (0.059) [-0.10, 0.13] -0.8		
BFZ versus BF					
*p-*value Difference (SE) 95% CI % reduction					0.348 -0.06 (0.059) [-0.17, 0.06] 3.0
BFZ versus BZ					
*p-*value Difference (SE) 95% CI % reduction					0.232 -0.07 (0.059) [-0.19, 0.05] 3.8
Interproximal Mean BOP					
Baseline					
n	53	51	52	50	52
Mean	0.109	0.104	0.156	0.099	0.113
(SD)	0.1519	0.1244	0.1990	0.1270	0.1304
Week 12					
n	52	50	52	50	50
LSmean (SE)	0.051 (0.0095)	0.039 (0.0097)	0.052 (0.0095)	0.019 (0.0097)	0.030 (0.0096)
Treatment groups versus B					
*p*-value		0.414	0.911	0.022	0.131
Difference (SE)		-0.011 (0.0135)	0.002 (0.0134)	-0.031 (0.0135)	-0.020 (0.0135)
95% CI		[-0.038, 0.016]	[-0.025, 0.028]	[-0.058, -0.005]	[-0.047, 0.006]
% reduction		21.9	-3.0	61.7	40.5
Brush/Rinse groups versus BF					
*p-*value Difference (SE) 95% CI % reduction		0.141 0.020 (0.0136) [-0.007, 0.047] -104.2	0.017 0.033 (0.0136) [0.006, 0.060] -169.2		
BFZ versus BF					
*p-*value Difference (SE) 95% CI % reduction					0.433 0.011 (0.0137) [-0.016, 0.038] -55.5
BFZ versus BZ					
*p-*value Difference (SE) 95% CI % reduction					0.107 -0.022 (0.0136) [-0.049, 0.005] 42.2

*B* Brush Only, *BA* Brush/Rinse (EO alcohol-containing mouthrinse), *BF* Brush/Floss, *BFZ* Brush/Floss/Rinse (EO non-alcohol containing mouthrinse), *BZ* Brush/Rinse (EO non-alcohol containing mouthrinse), *CI* confidence interval, *LSmean* least square means, *SD* standard deviation, *SE* standard error

All *p*-values, model-based estimated means (LSmeans), and standard errors were based on analysis of covariate with term for treatment and baseline as a covariate

### Secondary efficacy endpoint: whole mouth mean TPI and MGI at Week 4 and Week 12

The three groups using a mouthrinse all showed a statistically significant reduction in whole mouth mean TPI compared to the B group at Weeks 4 and 12. The BF group was not statistically significantly different from the B group for Week 4 or Week 12. Compared to BF, BA statistically significantly reduced whole mouth mean TPI at Week 4 (30.8%, *p* < 0.001) and Week 12 (37.6%, *p* < 0.001), and BZ statistically significantly reduced whole mouth mean TPI at Week 4 (21.0%, *p* < 0.001) and Week 12 (25.9%, *p* < 0.001). The BFZ group reduced TPI vs. BF at Week 4 (20.1%, *p* < 0.001) and Week 12 (23.7%, *p* < 0.001). The BFZ group was not statistically significantly different from BZ at Week 4 (*p* = 0.695) or Week 12 (*p* = 0.318) (Table 7).

**Table 7:** Whole mouth mean Turesky Plaque Index (TPI) at Baseline, Weeks 4, 12

Group	B	BA	BZ	BF	BFZ
Baseline					
n	53	51	52	50	52
Mean	3.07	2.98	3.00	3.13	3.03
(SD)	0.402	0.468	0.364	0.407	0.432
Week 4					
N	53	51	52	50	52
Lsmean (SE)	2.91 (0.046)	1.98 (0.047)	2.26 (0.046)	2.86 (0.047)	2.29 (0.046)
Treatment groups versus B					
*p*-value		< 0.001	< 0.001	0.430	< 0.001
Difference (SE)		-0.93 (0.065)	-0.65 (0.065)	-0.05 (0.066)	-0.63 (0.065)
95% CI		[-1.06, -0.80]	[-0.78, -0.52]	[-0.18, 0.08]	[-0.76, -0.50]
% reduction		32.0	22.4	1.80	21.5
Brush/Rinse groups versus BF Superiority					
*p*-value		< 0.001	< 0.001		
Difference (SE)		-0.88 (0.067)	-0.60 (0.066)		
95% CI		[-1.01, -0.75]	[-0.73, -0.47]		
% reduction		30.8	21.0		
BFZ versus BF Superiority					
*p*-value					< 0.001
Difference (SE)					-0.58 (0.066)
95% CI					[-0.71, -0.45]
% reduction					20.1
BFZ versus BZ					
*p*-value					0.695
Difference (SE)					0.03 (0.065)
95% CI					[-0.10, 0.15]
% reduction					-1.1
Week 12					
N	52	50	52	50	50
Lsmean (SE)	2.82 (0.045)	1.80 (0.046)	2.14 (0.045)	2.89 (0.046)	2.21 (0.046)
Treatment groups versus B					
*p*-value		< 0.001	< 0.001	0.229	< 0.001
Difference (SE)		-1.01 (0.064)	-0.67 (0.064)	0.08 (0.064)	-0.61 (0.064)
95% CI		[-1.14, -0.88]	[-0.80, -0.55]	[-0.05, 0.20]	[-0.73, -0.48]
% reduction		35.9	23.9	-2.8	21.6
Brush/Rinse groups versus BF Superiority					
*p*-value		< 0.001	< 0.001		
Difference		-1.09 (0.065)	-0.75 (0.065)		
95% CI		[-1.22, -0.96]	[-0.88, -0.62]		
% reduction		37.6	25.9		
BFZ versus BF Superiority					
*p*-value					< 0.001
Difference (SE)					-0.69 (0.065)
95% CI					[-0.81, -0.56]
% reduction					23.7
BFZ versus BZ Superiority					
*p*-value					0.318
Difference (SE)					0.06 (0.064)
95% CI					[-0.06, 0.19]
% reduction					-3.0

*B* Brush Only, *BA* Brush/Rinse (EO alcohol-containing mouthrinse), *BF* Brush/Floss, *BFZ* Brush/Floss/Rinse (EO non-alcohol containing mouthrinse), *BZ* Brush/Rinse (EO non-alcohol containing mouthrinse), *CI* confidence interval, *LSmean *least square means, *SD *standard deviation, *SE *standard error

All *p*-values, model-based estimated means (LSmeans), and standard errors were based on a mixed effects model for repeated measures analysis (MMRM), with the fixed effects including treatment, visit, and treatment by visit interaction; baseline as a covariate; and baseline by visit interaction

The three groups using a mouthrinse showed statistically significant reductions in whole mouth mean MGI compared to the B group at Weeks 4 and 12. The BF group reduced MGI by 6.7% (*p* = 0.016) at Week 4 but was not statistically significantly different from B at Week 12 (*p* = 0.076). Compared to BF, BA statistically significantly reduced whole mouth mean MGI at Week 4 (29.7%, *p* < 0.001) and Week 12 (42.6%, *p* < 0.001) and BZ statistically significantly reduced whole mouth mean MGI at Week 4 (30.3%, *p* < 0.001) and Week 12 (40.1%, *p* < 0.001). The BFZ group reduced MGI vs. BF at Week 4 (31.8%, *p* < 0.001) and Week 12 (38.1%, *p* < 0.001). The BFZ group was not statistically significantly different from BZ at Week 4 (*p* = 0.626) or Week 12 (*p* = 0.541) (Table 8).

**Table 8:** Whole mouth mean Modified Gingival Index (MGI) at Baseline, Weeks 4, 12

Group	B	BA	BZ	BF	BFZ
Baseline					
n	53	51	52	50	52
Mean	2.56	2.56	2.65	2.58	2.63
(SD)	0.307	0.320	0.288	0.270	0.261
Week 4					
N	53	51	52	50	52
LSmean (SE)	2.58 (0.050)	1.69 (0.051)	1.68 (0.050)	2.41 (0.051)	1.64 (0.050)
Treatment groups versus B					
*p*-value		< 0.001	< 0.001	0.016	< 0.001
Difference (SE)		-0.89 (0.071)	-0.90 (0.071)	-0.17 (0.071)	-0.94 (0.071)
95% CI		[-1.03, -0.75]	[-1.04, -0.76]	[-0.31, -0.03]	[-1.08, -0.80]
% reduction		34.4	35.0	6.7	36.3
Brush/Rinse groups versus BF Superiority					
*p*-value		< 0.001	< 0.001		
Difference (SE)		-0.72 (0.072)	-0.73 (0.072)		
95% CI		[-0.86, -0.57]	[-0.87, -0.59]		
% reduction		29.7	30.3		
BFZ group versus BF Superiority					
*p*-value					< 0.001
Difference (SE)					-0.77 (0.072)
95% CI					[-0.91, -0.62]
% reduction					31.8
BFZ versus BZ					
*p*-value					0.626
Difference (SE)					-0.03 (0.071)
95% CI					[-0.17, 0.10]
% reduction					2.10
Week 12					
N	52	50	52	50	50
LSmean (SE)	2.52 (0.054)	1.37 (0.055)	1.43 (0.054)	2.38 (0.055)	1.47 (0.055)
Treatment groups versus B					
*p*-value		< 0.001	< 0.001	0.076	< 0.001
Difference		-1.15 (0.077)	-1.09 (0.076)	-0.14 (0.077)	-1.04 (0.077)
95% CI		[-1.30, -1.00]	[-1.24, -0.94]	[-0.29, 0.01]	[-1.19, -0.89]
% reduction		45.7	43.3	5.4	41.5
Brush/Rinse groups versus BF Superiority					
*p*-value		< 0.001	< 0.001		
Difference (SE)		-1.01 (0.077)	-0.95 (0.077)		
95% CI		[-1.17, -0.86]	[-1.11, -0.80]		
% reduction		42.6	40.1		
BFZ versus BF Superiority					
*p*-value					< 0.001
Difference (SE)					-0.91 (0.077)
95% CI					[-1.06, -0.76]
% reduction					38.1
BFZ versus BZ Superiority					
*p*-value					0.541
Difference (SE)					0.05 (0.077)
95% CI					[-0.10, 0.20]
% reduction					-3.3

*B* Brush Only, *BA *Brush/Rinse (EO alcohol-containing mouthrinse), *BF *Brush/Floss, *BFZ *Brush/Floss/Rinse (EO non-alcohol containing mouthrinse), *BZ *Brush/Rinse (EO non-alcohol containing mouthrinse), *CI *confidence interval, *LSmean *least square means, *SD *standard deviation, *SE *standard error

All *p*-values, model-based estimated means (LSmeans), and standard errors were based on a mixed effects model for repeated measures analysis (MMRM), with the fixed effects including treatment, visit, and treatment by visit interaction; baseline as a covariate; and baseline by visit interaction

### Secondary efficacy endpoint: whole mouth mean EBI and percent bleeding sites at Week 4 and Week 12

All test groups showed statistically significant reductions in whole mouth mean EBI compared to the B group at Week 4 and Week 12 (*p* < 0.001 for each comparison). Both BA and BZ provided statistically significant reductions in EBI compared to BF (*p* < 0.001) at Week 4 and Week 12. In addition, the BFZ group provided a statistically significant reduction in EBI compared to BF (*p* < 0.001) at Weeks 4 and 12, but it was not statistically significantly different from BZ at Week 4 (*p* = 0.539) or Week 12 (*p* = 0.426) (Table 9).

**Table 9:** Whole mouth mean Expanded Bleeding Index (EBI) at Baseline, Weeks 4, 12

Group	B	BA	BZ	BF	BFZ
Baseline					
n	53	51	52	50	52
Mean	0.307	0.304	0.366	0.343	0.319
(SD)	0.1868	0.1695	0.1946	0.1741	0.1856
Week 4					
N	53	51	52	50	52
LSmean (SE)	0.229 (0.0097)	0.079 (0.0099)	0.076 (0.0099)	0.168 (0.0100)	0.067 (0.0098)
Treatment groups versus B					
*p*-value		< 0.001	< 0.001	< 0.001	< 0.001
Difference (SE)		-0.150 (0.0139)	-0.153 (0.0139)	-0.061 (0.0140)	-0.162 (0.0138)
95% CI		[-0.177, -0.123]	[-0.180, -0.126]	[-0.089, -0.034]	[-0.189, -0.134]
% reduction		65.6	66.9	26.7	70.7
Brush/Rinse groups versus BF Superiority					
*p*-value		< 0.001	< 0.001		
Difference (SE)		-0.089 (0.0141)	-0.092 (0.0140)		
95% CI		[-0.117, -0.061]	[-0.120, -0.064]		
% reduction		53.1	54.9		
BFZ versus BF Superiority					
*p*-value					< 0.001
Difference (SE)					-0.100 (0.0140)
95% CI					[-0.128, -0.073]
% reduction					60.0
BFZ versus BZ					
*p*-value					0.539
Difference (SE)					-0.009 (0.0139)
95% CI					[-0.036, 0.019]
% reduction					11.3
Week 12					
N	52	50	52	50	50
LSmean (SE)	0.257 (0.0121)	0.086 (0.0123)	0.081 (0.0121)	0.185 (0.0123)	0.067 (0.0123)
Treatment group versus B					
*p*-value		< 0.001	< 0.001	< 0.001	< 0.001
Difference		-0.170 (0.0172)	-0.176 (0.0172)	-0.071 (0.0173)	-0.190 (0.0172)
95% CI		[-0.204, -0.137]	[-0.210, -0.142]	[-0.105, -0.037]	[-0.224, -0.156]
% reduction		66.4	68.5	27.8	73.9
Brush/Rinse groups versus BF Superiority					
*p*-value		< 0.001	< 0.001		
Difference (SE)		-0.099 (0.0174)	-0.104 (0.0173)		
95% CI		[-0.134, -0.065]	[-0.139, -0.070]		
% reduction		53.5	56.4		
BFZ versus BF Superiority					
*p*-value					< 0.001
Difference (SE)					-0.118 (0.0174)
95% CI					[-0.153, -0.084]
% reduction					63.8
BFZ versus BZ Superiority					
*p*-value					0.426
Difference (SE)					-0.014 (0.0173)
95% CI					[-0.048, 0.020]
% reduction					17.1

*B* Brush Only, *BA* Brush/Rinse (EO alcohol-containing mouthrinse), *BF *Brush/Floss, *BFZ *Brush/Floss/Rinse (EO non-alcohol containing mouthrinse), *BZ *Brush/Rinse (EO non-alcohol containing mouthrinse), *CI *confidence interval, *LSmean *least square means, *SD *standard deviation, *SE *standard error

All *p*-values, model-based estimated means (LSmeans), and standard errors were based on a mixed effects model for repeated measures analysis (MMRM), with the fixed effects including treatment, visit, and treatment by visit interaction; baseline as a covariate; and baseline by visit interaction

Results were similar for the secondary endpoint of whole mouth percent bleeding sites based on EBI after 4 and 12 weeks of product use. All test groups showed statistically significant reductions in percent bleeding sites compared to the B group at Week 4 and Week 12 (*p* < 0.001 for each comparison). Both BA and BZ provided statistically significant reductions in percent bleeding sites compared to BF (*p* < 0.001) at Week 4 and Week 12. In addition, BFZ group provided a statistically significant reduction in percent bleeding sites compared to BF (*p* < 0.001), and it was not statistically significantly different from BZ at Week 4 (*p* = 0.498) or Week 12 (*p* = 0.419) (Table 10).

**Table 10:** Whole mouth percent bleeding sites (based on EBI) at Weeks 4 and 12

Group	B	BA	BZ	BF	BFZ
Baseline					
n	53	51	52	50	52
Mean	24.9	24.8	28.8	26.8	25.4
(SD)	11.52	11.54	12.15	10.43	11.08
Week 4					
N	53	51	52	50	52
LSmean (SE)	19.4 (0.73)	6.5 (0.74)	6.6 (0.74)	14.6 (0.75)	5.9 (0.73)
Treatment groups versus B					
*p*-value		< 0.001	< 0.001	< 0.001	< 0.001
Difference (SE)		-12.9 (1.04)	-12.8 (1.04)	-4.8 (1.04)	-13.5 (1.03)
95% CI		[-15.0, -10.9]	[-14.9, -10.8]	[-6.9, -2.8]	[-15.6, -11.5]
% reduction		66.6	66.1	24.8	69.7
Brush/Rinse groups versus Brush/Floss Superiority					
*p*-value		< 0.001	< 0.001		
Difference (SE)		-8.1 (1.05)	-8.0 (1.05)		
95% CI		[-10.2, -6.0]	[-10.1, -5.9]		
% reduction		55.5	54.9		
BFZ versus BF Superiority					
*p*-value					< 0.001
Difference (SE)					-8.7 (1.05)
95% CI					[-10.8, -6.7]
% reduction					59.7
BFZ versus BZ					
*p*-value					0.498
Difference (SE)					-0.7 (1.04)
95% CI					[-2.8, 1.3]
% reduction					10.7
Week 12					
N	52	50	52	50	50
LSmean (SE)	21.2 (0.90)	7.4 (0.92)	7.1 (0.90)	16.6 (0.92)	6.1 (0.91)
Treatment groups versus B					
*p*-value		< 0.001	< 0.001	< 0.001	< 0.001
Difference (SE)		-13.8 (1.28)	-14.1 (1.28)	-4.7 (1.28)	-15.2 (1.28)
95% CI		[-16.4, -11.3]	[-16.6, -11.6]	[-7.2, -2.1]	[-17.7, -12.6]
% reduction		65.2	66.5	22.0	71.4
Brush/Rinse groups versus BF Superiority					
*p*-value		< 0.001	< 0.001		
Difference (SE)		-9.2 (1.30)	-9.5 (1.29)		
95% CI		[-11.7, -6.6]	[-12.0, -6.9]		
% reduction		55.4	57.1		
BFZ versus BF Superiority					
*p*-value					< 0.001
Difference (SE)					-10.5 (1.30)
95% CI					[-13.0, -7.9]
% reduction					63.4
BFZ versus BZ Superiority					
*p*-value					0.419
Difference (SE)					-1.0 (1.29)
95% CI					[-3.6, 1.5]
% reduction					14.7

*B* Brush Only, *BA *Brush/Rinse (EO alcohol-containing mouthrinse), *BF *Brush/Floss, *BFZ *Brush/Floss/Rinse (EO non-alcohol containing mouthrinse), *BZ *Brush/Rinse (EO non-alcohol containing mouthrinse), *CI *confidence interval, *LSmean *least square means, *SD *standard deviation, *SE *standard error

All *p*-values, model-based estimated means (LSmeans), and standard errors were based on a mixed effects model for repeated measures analysis (MMRM), with the fixed effects including treatment, visit, and treatment by visit interaction; baseline as a covariate; and baseline by visit interaction

### Secondary efficacy endpoint: whole mouth mean pocket depth and BOP at week 12

All test groups showed statistically significant reductions in mean pocket depth compared to the B group at Week 12 (*p* < 0.001 for each comparison). There were no statistically significant differences in mean pocket depth for BA or BZ when compared with BF group. The BFZ group was not statistically significantly different from BF (*p* = 0.512) or BZ (*p* = 0.203) for mean pocket depth at Week 12. Only the BF group had statistically significantly reduced BOP (*p* = 0.028) compared to the B group. BF significantly reduced BOP vs. BZ (*p* = 0.033). The BFZ group was not statistically significantly different from BF (*p* = 0.524) or BZ (*p* = 0.132) for mean BOP at Week 12 (Table 6).

### Secondary efficacy endpoint: marginal mean TPI, MGI and EBI at week 4 and week 12

The three groups using a mouthrinse all showed a statistically significant reduction in marginal mean TPI compared to the B group at Weeks 4 and 12. The BF group was not statistically significantly different from B for Week 4 (*p* = 0.225) or Week 12 (*p* = 0.447). Compared to BF, BA statistically significantly reduced marginal mean TPI at Week 4 (40.4%, *p* < 0.001) and Week 12 (49.4%, *p* < 0.001), and BZ statistically significantly reduced TPI at Week 4 (30.3%, *p* < 0.001) and Week 12 (38.8%, *p* < 0.001). The BFZ group statistically significantly reduced TPI vs. BF at Week 4 (30.0%, *p* < 0.001) and Week 12 (36.4%, *p* < 0.001). The BFZ group was not statistically significantly different from BZ at Week 4 (*p* = 0.934) or Week 12 (*p* = 0.470).

The three groups using mouthrinses all showed statistically significant reductions in marginal mean MGI compared to the B group at Weeks 4 and 12. The BF group reduced MGI by 8.1% (*p* = 0.011) at Week 4 but was not statistically significant at Week 12 (*p* = 0.072). Compared to BF, BA statistically significantly reduced marginal mean MGI at Week 4 (34.3%, *p* < 0.001) and Week 12 (49.0%, *p* < 0.001) and BZ statistically significantly reduced mean MGI at Week 4 (34.9%, *p* < 0.001) and Week 12 (46.0%, *p* < 0.001). The BFZ group statistically significantly reduced MGI vs. BF at Week 4 (36.0%, *p* < 0.001) and Week 12 (43.0%, *p* < 0.001). The BFZ group was not statistically significantly different from BZ at Week 4 (*p* = 0.766) or Week 12 (*p* = 0.413).

All test groups showed statistically significant reductions in marginal mean EBI compared to the B group at Week 4 and Week 12. Both BA and BZ provided statistically significant reductions in EBI compared to BF at Week 4 (*p* < 0.001 for each comparisons) and Week 12 (*p* < 0.001 for each comparison). In addition, the BFZ group provided a statistically significant reduction in EBI compared to BF at Weeks 4 (*p* < 0.001) and 12 (*p* < 0.001). BFZ was not statistically significantly different from BZ at Week 4 (*p* = 0.918) or Week 12 (*p* = 0.483). Marginal mean TPI, MGI and EBI are shown in Table 11.

**Table 11:** Marginal mean Turesky Plaque Index (TPI), Modified Gingival Index (MGI) and Expanded Bleeding Index (EBI) at Baseline, Week 4 and Week 12

Group	B	BA	BZ	BF	BFZ
Marginal Mean Turesky Plaque Index (TPI)					
Baseline					
n	53	51	52	50	52
Mean	2.80	2.68	2.73	2.85	2.74
(SD)	0.473	0.610	0.462	0.508	0.553
Week 4					
N	53	51	52	50	52
LSmean (SE)	2.59 (0.060)	1.48 (0.062)	1.73 (0.061)	2.49 (0.062)	1.74 (0.061)
Treatment groups versus B					
*p*-value		< 0.001	< 0.001	0.225	< 0.001
Difference (SE)		-1.11 (0.086)	-0.86 (0.086)	-0.11 (0.086)	-0.85 (0.086)
95% CI		[-1.28, -0.94]	[-1.03, -0.69]	[-0.28, 0.07]	[-1.02, -0.68]
% reduction		42.8	33.2	4.1	32.9
Brush/Rinse groups versus BF Superiority					
*p*-value		< 0.001	< 0.001		
Difference (SE)		-1.01 (0.088)	-0.75 (0.087)		
95% CI		[-1.18, -0.83]	[-0.93, -0.58]		
% reduction		40.4	30.3		
BFZ versus BF Superiority					
*p*-value					< 0.001
Difference (SE)					-0.75 (0.087)
95% CI					[-0.92, -0.58]
% reduction					30.0
BFZ versus BZ					
*p*-value					0.934
Difference (SE)					0.01 (0.086)
95% CI					[-0.16, 0.18]
% reduction					-0.4
Week 12					
N	52	50	52	50	50
LSmean (SE)	2.51 (0.060)	1.31 (0.061)	1.58 (0.060)	2.58 (0.061)	1.64 (0.061)
Treatment groups versus B					
*p*-value		< 0.001	< 0.001	0.447	< 0.001
Difference (SE)		-1.21 (0.085)	-0.94 (0.085)	0.07 (0.085)	-0.87 (0.085)
95% CI		[-1.38, -1.04]	[-1.10, -0.77]	[-0.10, 0.23]	[-1.04, -0.71]
% reduction		48.1	37.2	-2.6	34.8
Brush/Rinse groups versus BF Superiority					
*p*-value		< 0.001	< 0.001		
Difference (SE)		-1.27 (0.087)	-1.00 (0.086)		
95% CI		[-1.44, -1.10]	[-1.17, -0.83]		
% reduction		49.4	38.8		
BFZ versus BF Superiority					
*p*-value					< 0.001
Difference (SE)					-0.94 (0.086)
95% CI					[-1.11, -0.77]
% reduction					36.4
BFZ versus BZ Superiority					
*p*-value					0.470
Difference (SE)					0.06 (0.085)
95% CI					[-0.11, 0.23]
% reduction					-3.9
Marginal Mean Modified Gingival Index (MGI)					
Baseline					
n	53	51	52	50	52
Mean	2.40	2.42	2.52	2.43	2.50
(SD)	0.366	0.375	0.366	0.332	0.332
Week 4					
N	53	51	52	50	52
LSmean	2.44 (0.054)	1.47 (0.055)	1.46 (0.055)	2.24 (0.056)	1.43 (0.055)
Treatment groups versus B					
*p*-value		< 0.001	< 0.001	0.011	< 0.001
Difference (SE)		-0.97 (0.077)	-0.98 (0.077)	-0.20 (0.078)	-1.00 (0.077)
95% CI		[-1.12, -0.81]	[-1.13, -0.83]	[-0.35, -0.05]	[-1.16, -0.85]
% reduction		39.6	40.2	8.1	41.2
Brush/Rinse groups versus BF Superiority					
*p*-value		< 0.001	< 0.001		
Difference (SE)		-0.77 (0.078)	-0.78 (0.078)		
95% CI		[-0.92, -0.61]	[-0.94, -0.63]		
% reduction		34.3	34.9		
BFZ versus BF Superiority					
*p*-value					< 0.001
Difference (SE)					-0.81 (0.078)
95% CI					[-0.96, -0.65]
% reduction					36.0
BFZ versus BZ					
*p*-value					0.766
Difference (SE)					-0.02 (0.077)
95% CI					[-0.18, 0.13]
% reduction					1.6
Week 12					
N	52	50	52	50	50
LSmean (SE)	2.38 (0.057)	1.14 (0.058)	1.20 (0.057)	2.23 (0.058)	1.27 (0.058)
Treatment groups versus B					
*p*-value		< 0.001	< 0.001	0.072	< 0.001
Difference (SE)		-1.24 (0.082)	-1.17 (0.081)	-0.15 (0.082)	-1.11 (0.082)
95% CI		[-1.40, -1.08]	[-1.33, -1.01]	[-0.31, 0.01]	[-1.27, -0.94]
% reduction		52.2	49.3	6.2	46.5
Brush/Rinse groups versus BF Superiority					
*p*-value		< 0.001	< 0.001		
Difference (SE)		-1.09 (0.082)	-1.03 (0.082)		
95% CI		[-1.26, -0.93]	[-1.19, -0.86]		
% reduction		49.0	46.0		
BFZ versus BF Superiority					
*p*-value					< 0.001
Difference					-0.96 (0.082)
95% CI					[-1.12, -0.80]
% reduction					43.0
BFZ versus BZ Superiority					
*p*-value					0.413
Difference (SE)					0.07 (0.082)
95% CI					[-0.09, 0.23]
% reduction					-5.5
Marginal Mean Expanded Bleeding Index (EBI)					
Baseline					
n	53	51	52	50	52
Mean	0.312	0.319	0.364	0.344	0.335
(SD)	0.1929	0.1762	0.1928	0.1897	0.2029
Week 4					
N	53	51	52	50	52
LSmean	0.260 (0.0123)	0.107 (0.0125)	0.104 (0.0124)	0.225 (0.0126)	0.106 (0.0124)
Treatment groups versus B					
*p*-value		< 0.001	< 0.001	0.045	< 0.001
Difference (SE)		-0.153 (0.0175)	-0.156 (0.0175)	-0.035 (0.0176)	-0.155 (0.0175)
95% CI		[-0.187, -0.118]	[-0.191, -0.122]	[-0.070, -0.001]	[-0.189, -0.120]
% reduction		58.7	60.1	13.6	59.4
Brush/Rinse groups versus BF Superiority					
*p*-value		< 0.001	< 0.001		
Difference (SE)		-0.117 (0.0178)	-0.121 (0.0177)		
95% CI		[-0.152, -0.082]	[-0.156, -0.086]		
% reduction		52.2	53.8		
BFZ versus BF Superiority					
*p*-value					< 0.001
Difference (SE)					-0.119 (0.0177)
95% CI					[-0.154, -0.084]
% reduction					53.0
BFZ versus BZ					
*p*-value					0.918
Difference					0.002 (0.0175)
95% CI					[-0.033, 0.036]
% reduction					-1.8
Week 12					
N	52	50	52	50	50
LSmean (SE)	0.288 (0.0142)	0.102 (0.0144)	0.117 (0.0142)	0.226 (0.0145)	0.103 (0.0144)
Treatment groups versus B					
*p*-value		< 0.001	< 0.001	0.003	< 0.001
Difference (SE)		-0.186 (0.0202)	-0.171 (0.0201)	-0.062 (0.0203)	-0.185 (0.0202)
95% CI		[-0.226, -0.146]	[-0.211, -0.132]	[-0.102, -0.022]	[-0.225, -0.146]
% reduction		64.5	59.4	21.4	64.4
Brush/Rinse groups versus BF Superiority					
*p*-value		< 0.001	< 0.001		
Difference (SE)		-0.124 (0.0205)	-0.110 (0.0203)		
95% CI		[-0.164, -0.084]	[-0.150, -0.070]		
% reduction		54.8	48.4		
BFZ versus BF Superiority					
*p*-value					< 0.001
Difference (SE)					-0.124 (0.0204)
95% CI					[-0.164, -0.084]
% reduction					54.6
BFZ versus BZ Superiority					
*p*-value					0.483
Difference (SE)					-0.014 (0.0203)
95% CI					[-0.054, 0.026]
% reduction					12.2

*B* Brush Only, *BA* Brush/Rinse (EO alcohol-containing mouthrinse), *BF *Brush/Floss, *BFZ *Brush/Floss/Rinse (EO non-alcohol containing mouthrinse), *BZ *Brush/Rinse (EO non-alcohol containing mouthrinse), *CI *confidence interval, *LSmean *least square means, *SD *standard deviation, *SE *standard error

All *p*-values, model-based estimated means (LSmeans), and standard errors were based on a mixed effects model for repeated measures analysis (MMRM), with the fixed effects including treatment, visit, and treatment by visit interaction; baseline as a covariate; and baseline by visit interaction

### Safety

There were 15 subjects with at least one treatment-related AE in this study. Two AEs were unrelated to the treatment: an aphthous ulcer (B group) was mild, resolved without treatment and the subject completed the study; and a urinary tract infection (BF group), which was mild, required medication and the subject withdrew from the study. In the mouthrinse groups, there were four subjects in the BA group who experienced oral mucosal exfoliation. Each was a single episode, classified as mild severity and probably or very likely related to treatment. Each was resolved without treatment and the subjects completed the study. Four subjects in the BZ group experienced oral mucosal exfoliation. Three were classified as mild; one as moderate. Each was a single episode and probably or very likely related to the treatment. Each was resolved without treatment and the subjects completed the study. The BFZ group had six subjects who experienced oral mucosal exfoliation and one subject who experienced an aphthous ulcer. Five incidents of exfoliation and the one aphthous ulcer were classified as mild; one exfoliation incident was classified as moderate. All AEs were single episodes; all resolved with no treatment and the subjects completed the study. No deaths and no serious treatment emergent AEs (TEAEs) were reported. No TEAEs resulted in subject withdrawal from the trial.

## Discussion

The purpose of this 12-week clinical trial was to investigate the effects of various supervised combinations of mechanical (brushing and flossing) and chemotherapeutic regimens, which included four EOs non-alcohol and alcohol-containing mouthrinses, compared to brushing only or brushing and flossing, in the prevention and reduction of plaque, gingivitis and gingival bleeding. To address patient needs, this information is valuable for professionals who recommend non-alcohol containing mouthrinses. The primary endpoints revealed that groups using the EO mouthrinses, non-alcohol or alcohol-containing, with or without flossing, twice daily demonstrated statistically significant reductions in gingivitis and supragingival plaque after 12 weeks compared to brushing alone. This was observed in all area-specific parameters, including interproximal, marginal and whole mouth regions. Similar findings for alcohol-containing EO mouthrinses were reported in Milleman et al. 2022 [7] and Bosma et al. 2022 [13].

In this study, the use of dental floss and brushing (BF) did not reduce supragingival plaque compared to B, but it did demonstrate statistically significantly reductions in interproximal EBI at 4 and 12 weeks and percent bleeding sites based on EBI, although not as effectively as any of the mouthrinsing regimens. While the mechanical action of flossing disrupts the plaque mass leading to short term reductions, this effect does not appear to be sustained over time thus preventing statistically significant plaque reductions to be observed at 8–18 h after flossing [7, 13]. The absence of an effect on interproximal supragingival plaque in the BF group has been previously reported in a study of a similar design [7]. In that study, the groups assigned to regimens incorporating a chemotherapeutic EO alcohol-containing mouthrinse with or without floss achieved a sustained reduction in supragingival plaque at all timepoints (4 and 12 weeks) throughout the study. The results of this current study not only confirm these findings for the EO alcohol-containing mouthrinse but also extend this effect to the EO non-alcohol containing mouthrinse. Both the alcohol and non-alcohol containing formulations tested in these studies contain a fixed combination of menthol, thymol, eucalyptol, and methyl salicylate, which disrupts the bacterial cell wall, leading to a reduction in bacterial regrowth and, consequently, enhanced control of supragingival plaque [9, 13–18].

In the current study, the alcohol-containing mouthrinse and non-alcohol containing mouthrinse reduced plaque by 30.8% and 18.2%, respectively, compared to brushing alone. However, gingivitis (MGI) and bleeding (EBI) were similar between the two regimens. A prior study by Lynch et al. reported that, in conjunction with mechanical oral hygiene (MOH), EO alcohol-containing and EO non-alcohol containing mouthrinses resulted in similar reductions in gingivitis and plaque at one, three and six months when compared to MOH only (MOH was brushing and, if part of the subject’s usual oral care regimen, continued use of an interdental cleaning device) [9]. One difference between the Lynch et al. study and the present study is the virtual supervision in the present study. While plaque is a contributor to patient oral health, the true measure of health is the absence of gingivitis. In the present study, subjects who used EO mouthrinses, either with or without alcohol, showed statistically significant reduction in gingivitis (39.0% and 36.9%, respectively) and plaque (30.8% and 18.2%, respectively), compared to those brushing alone at Week 12. Our study highlights that the non-alcohol containing product, when used twice daily with or without floss, remains statistically significantly more effective in the control of supragingival plaque and other parameters compared to brushing alone or brushing and flossing.

While secondary endpoints, the whole mouth results for MGI and EBI point to reductions in areas where floss cannot work due to its targeted mechanical application in the interproximal space. The addition of mouthrinse to hygiene regimens allows the chemotherapeutic effect to occur in the whole mouth as evidenced by the whole mouth results where the BFZ group provided a statistically significant reduction in EBI compared to BF at Week 4 (60%, *p* < 0.001) and Week 12 (63.8%, *p* < 0.001,) and the BFZ group reduced MGI vs. BF at Week 4 (31.8%, *p* < 0.001) and Week 12 (38.1%, *p* < 0.001). In this study, probing depth and BOP were measured at baseline and after 12 weeks of product use. All test groups showed statistically significant reductions in interproximal and whole mouth mean pocket depth compared to the B group. In addition, BOP was statistically significantly reduced for BF group compared to the B group by 55.7% (*p* = 0.028) for whole mouth. The BFZ group, while not statistically significant compared to the B group, resulted in a numerical difference of 39.4% (*p* = 0.120). Similarly, for interproximal BOP, statistically significant reductions were observed for the BF group compared to the B group at 61.7% (*p* = 0.022), while a numerical reduction of 40.5% (*p* = 0.131) was noted for the BFZ group compared to the B group. While not statistically significant for BFZ, this study was not designed to specifically examine floss effects on BOP and subjects were not recruited for this purpose. Nevertheless, BOP is an indicator of increased inflammation at these sites compared to non-bleeding sites and may serve as an earlier sign of gingivitis than erythema and edema [19]. A similar pattern was also seen for the floss groups in the Milleman et al. study [7]. It is hypothesized that floss provides deeper subgingival access compared to the chemical control from mouthrinsing alone.

A number of professional and governmental organizations, such as the National Health Service UK (NHS) [6], World Dental Federation (FDI) [20], and ADA [12] emphasize the importance of daily interdental cleaning in addition to toothbrushing for maintaining a healthy mouth through their website. The findings of this study highlight the significance of incorporating an EO mouthrinse as an important adjunct to an oral hygiene regimen that includes interdental cleaning. This information may help the practitioner determine tailored oral hygiene regimens that effectively address the specific dental needs of their patients, ultimately improving the quality of care provided.

### Limitations

The evaluation of disease severity was based solely on clinical assessments, without the use of radiographs to verify the absence of severe periodontitis. Since our cohort was pre-selected with specific criteria, it may not accurately represent the wider population with less disease or greater levels of disease. The study could not be double-blinded due to the inclusion of dental floss in some, but not all, of the treatment groups and to the differences in color and taste of the alcohol-containing and non-alcohol containing mouthwashes. To address these limitations and avoid bias in the current study, appropriate study design elements included having subjects use study products in specific designated areas in the study clinic; instructing subjects to not discuss the study products they were assigned with examiners; not allowing personnel dispensing the study products or supervising or observing their use to participate in the examination of subjects; and not allowing examiners and investigators into in the area where study products were administered. While subjects received flossing instruction, toothbrushing instruction was not provided and subjects continued their usual brushing practices. The study assessed only the effects of once-daily flossing with waxed floss after brushing rather than exploring various floss types, multiple flossing sessions per day, or flossing prior to brushing. It is also important to note that the investigation was limited to floss as the sole interdental cleaning device that was investigated in this trial.

## Conclusions

The twice-daily use of mouthrinses with a fixed combination of four essential oils (menthol, thymol, eucalyptol and methyl salicylate), either alcohol or non-alcohol containing, in conjunction with manual toothbrushing significantly reduced plaque, gingivitis, and bleeding at 4 and 12 weeks as compared to brushing alone. Furthermore, adding essential oil non-alcohol containing mouthrinse to the brushing and flossing regimen also significantly reduced plaque, gingivitis and bleeding compared to brushing and flossing alone. The recommendation of the addition of an essential oil non-alcohol containing mouthrinse to daily oral hygiene routines of brushing or brushing and flossing should be considered by the practitioner to aid supragingival plaque control and improve gingivitis prevention.

## Data Availability

The data sharing policy of JJC is available at https://www.janssen.com/clinical-trials/transparency. As noted on this site, requests for access to the study data can be submitted through Yale Open Data Access (YODA) Project site at http://yoda.yale.edu.

## Abbreviations

ADA: American Dental Association
ACM: Antiseptic EO alcohol-containing mouthrinse
AE: Adverse event
AFM: Antiseptic EO non-alcohol containing mouthrinse
ANOVA: Analysis of variance
B: Brush only
BA: Brush/rinse with EO alcohol-containing mouthrinse
BF: Brush/floss
BFZ: Brush/floss/rinse with EO non-alcohol containing mouthrinse
BOP: Bleeding on probing
BZ: Brush/rinse with EO non-alcohol containing mouthrinse
CI: Confidence interval
EBI: Expanded bleeding index
EO: Essential oils
FDI: World Dental Federation
ICH: International Council on Harmonisation
JJC: Johnson & Johnson Consumer
MGI: Modified gingival index
MMRM: Mixed effects model for repeated measures
MOH: Mechanical oral hygiene
NHS: National Health Service
SD: Standard deviation
SEM: Standard error of the mean
TEAE: Treatment emergent adverse event
TPI: Turesky Plaque Index
YODA: Yale Open Data Access
